# Obese Parturient: A Modern-Day Obstetric Challenge

**DOI:** 10.7759/cureus.39958

**Published:** 2023-06-04

**Authors:** Nehal Shah, Varshil Mehta

**Affiliations:** 1 Obstetrics and Gynecology, Lokmanya Tilak Municipal General Hospital, Mumbai, IND; 2 Genitourinary Medicine, Northwick Park Hospital, London, GBR

**Keywords:** high-risk obstetrics, lower segment cesarean section, preterm delivery, complication, super morbid obesity

## Abstract

Obesity has become a growing pandemic with a significant increase in incidence in recent years. The complications associated with pregnancy in obese patients can lead to increased morbidity and mortality in pregnant women. A 41-year-old morbidly obese female with primary hypertension and 32.4 weeks pregnant, presented with severe oligohydramnios, breech presentation, and a history of previous lower segment cesarean section (LSCS). The patient experienced abdominal pain, lower backache, and leaking per vaginal, and a decision was made to perform LSCS. Challenges were encountered during the procedure related to anesthesia management and the need for specialized equipment and additional assistants. A multidisciplinary approach was chosen for managing this patient with the special role of anesthetists. Intra-operative and post-operative management was crucial for a successful recovery. Obesity during pregnancy presents unique challenges for healthcare providers, and it is necessary to increase resources and prepare skilfully to manage these patients effectively.

## Introduction

According to the available literature, many nations across the globe have experienced a twofold or threefold rise in the incidence of obesity in the last three decades, which can be attributed to a sedentary lifestyle, urbanization, and a rise in the consumption of highly calorific processed foods [1].

This phenomenon impacts people of all social strata and age groups, including adolescents and the elderly. Consequently, an elevated number of obese females are becoming pregnant [2], resulting in a multitude of health problems and an increase in complications for both the mother and the baby including preeclampsia, gestational diabetes and hypertension, premature delivery, and spontaneous abortions [3,4]. Hence, it's important to take different steps to recognize and address patients with obesity issues and encourage weight loss strategies before pregnancy through methods like lifestyle modification, and medical or surgical management.

Here, we present a case of such a patient who was found to be a challenge for the obstetricians due to her obesity, yet managed well following a lower segment cesarean section (LSCS) procedure.

## Case presentation

A morbidly obese female, in her early 40s and 32.4 weeks pregnant, with gravida 2, parity 1 (G_2_P_1_), and with a background of previous LSCS was referred to our tertiary center from a private maternity center due to potential risks involved with her delivery.

In her previous pregnancy, she developed gestational hypertension and was started on treatment, which was discontinued after delivery. It was a post-term pregnancy and hence, on admission, she was induced for labor. However, she had to undergo a successful cesarean section as there was fetal distress.

As per her records, in the current pregnancy, she weighed 131 kg (BMI: 51.17 kg/m^2^) before becoming pregnant. On her first visit during her 12th week of gestation, she was started on nifedipine 10 mg twice daily and 150 mg of aspirin due to moderately raised blood pressure by her private obstetrician and gynecologist. She was screened and was not found to have gestational diabetes and her urine dipstick was negative for proteins and glucose. The patient was followed up with antenatal clinic routinely thereafter. During her seventh-month follow-up, while her blood pressure was well-controlled with the continued use of antihypertensives, her weight had increased by 5 kg to 136 kg (BMI: 53.1 kg/m^2^), and thus, grading her into a super or extreme obese group (BMI: >50 kg/m^2^).

She presented with a six-hour history of abdominal pain and lower backache, as well as a three-hour history of preterm rupture of membrane. During the examination, it was challenging to assess her uterine size accurately, and it was difficult to determine if there was any scar tenderness due to her pendulous abdomen. The fetal heart rate was challenging to locate due to the same reason. Upon speculum examination, it was found that her cervix was 1.5 cm dilated with pre-term pre-labor rupture of membrane.

Ultrasonography showed a single live intra-uterine gestation with an approximate gestational age of 32 weeks and an estimated fetal weight of 2 kg. It also showed that the fetus was in a breech presentation with the placenta being placed anteriorly, and an amniotic fluid index (AFI) of 0-1 cm. Her observations were stable and other investigations including urine dipstick were within normal limits.

The patient was informed of the high risk associated with vaginal birth after cesarean section (VBAC), especially due to difficulty in monitoring labor for impending scar rupture secondarily to morbid obesity, she was advised LSCS. The patient understood and opted for LSCS. Patient was also given a single dose of intramuscular (IM) betamethasone 12 mg for fetal lung maturity.

Pre-operative management

All pre-operative consents were obtained in written form, including informed consent, high-risk consent, guarded fetomaternal consent for prolonged neonatal intensive care unit (NICU) admission, negative consent for VBAC, and impending intra-uterine fetal death (IUFD) consent. A multidisciplinary approach was chosen to manage this patient, with a special role for anesthesiologists. The anaesthesiologists conducted a detailed pre-anesthetic check-up and decided to administer combined spinal and epidural anesthesia. Two venous access points were secured by a senior anesthesiologist, and extra instruments were kept on hand, such as malleable and longer Deaver's retractors, Doyen's retractor with deeper blades, and an extra set of vulsellums. An extra operational theatre (OT) table was on standby if needed. Additional residents (trainees) were mobilized to assist with the surgery, and they were briefed about their roles as this was their first case of this kind. Pre-operative prophylactic antibiotics of 3 g of cefazolin were administered. After all the pre-operative prerequisites were met, the patient was taken to the operating room. The abdomen was cleaned with povidone-iodine half an hour before the procedure, and the area was cleaned again in the usual manner with special attention paid to the panniculus and groin area.

Intra-operative management 

**Figure 1:**
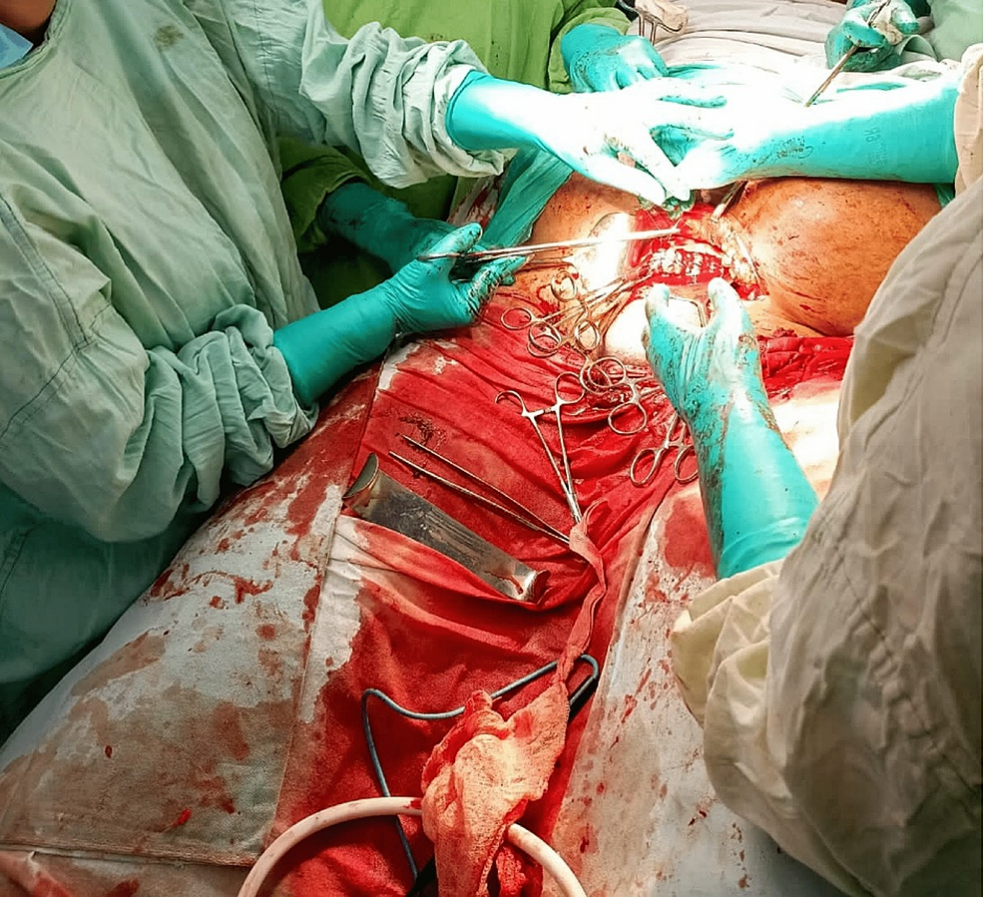
Intra-operative procedure of the patient.

Under spinal and epidural anesthesia, a decision was made to perform a low transverse incision. Two additional residents were required at the head end of the patient to retract the abdomen, which was done with two vulsellums held at the rectus sheath, with the ends of the vulsellum held by the assistants. Two others were required to retract the bladder at the leg end of the patient on either side of the table. The layers of the abdomen were opened with cautery to minimize bleeding (Figure 1). Omental adhesions were present and were separated by sharp dissection. After around 20 minutes of dissection, access was gained to the uterus, and a preterm male child weighing 2,000 g was delivered by breech extraction. The uterus was atonic and patient went into primary post-partum hemorrhage (PPH) for a short period with blood loss of approximately 710 mL, but once the tone was achieved with uterotonics (oxytocin and carboprost), PPH resolved.

Closure of the abdomen involved not closing the peritoneum. The rectus muscle was closed with Vicryl 2-0 after thorough checking for hemostasis to prevent hematoma formation. The rectus sheath was closed with Ethilon 1. Subcutaneous tissue was stitched in three layers with Vicryl 2-0, and the skin was closed with a vertical mattress using Ethilon 1-0. After the sterile dressing of the wound, the lower abdomen was strapped to the upper abdomen for better healing to avoid contamination due to moisture from the sweat under the panniculus. No drains were kept due to their questionable role in preventing wound infection.

Post-operative care

**Figure 2:**
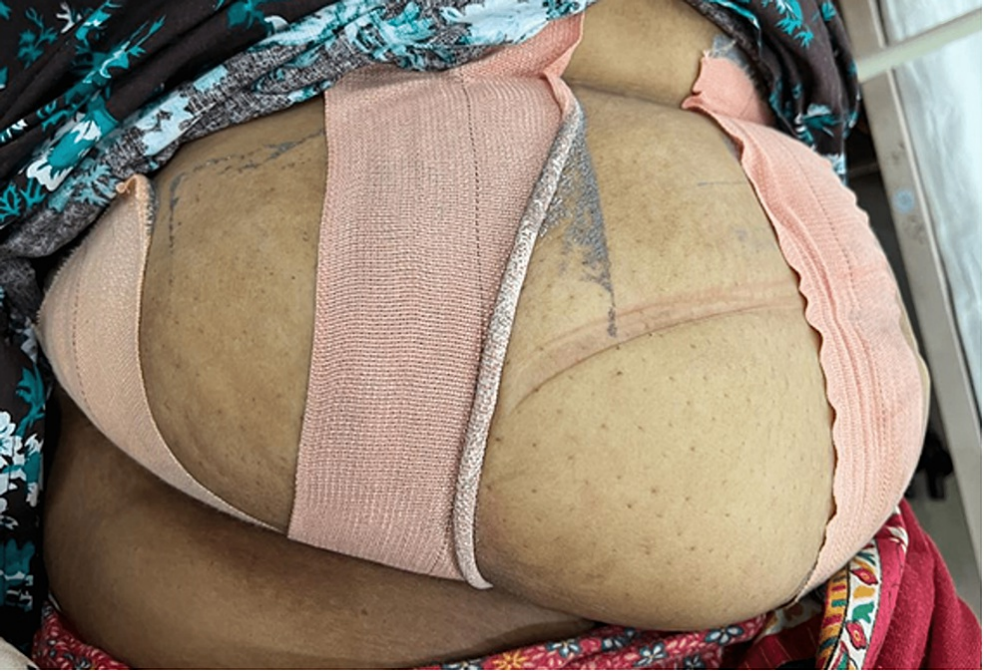
Post-operative care of the patient.

The patient was shifted to the ward on an O_2_ bed with an air mattress made available. Oxygen (O_2_) @ 4 L/min through Hudson's mask and intravenous (IV) fluids 2 mL/kg/h were also initiated. Intravenous (IV) antibiotics were started according to her weight, and anti-hypertensive medications were continued. The patient was made to wear compression stockings, and thromboprophylaxis with enoxaparin 80 mg once daily (OD) was initiated after 12 h. Vital signs were monitored at regular intervals, and early mobilization was encouraged. The dressing was checked first on post-operative day three which was found to be healthy followed by another sterile dressing done with strapping of the abdomen in a similar manner (Figure 2). She was also prescribed an abdominal belt in order to avoid the folding of her belly and sweating under panniculus. Patient was also counseled for wound care and personal hygiene maintenance and was also motivated for continuing lactation. Her post-partum care remained uneventful. She was given thorough knowledge regarding weight management interventions including the option of undergoing bariatric surgery before discharge. She was called for follow-up on post-operative day 10 and the wound was found to be healthy and healing. She was again followed up on day 14th and her sutures were removed.

## Discussion

Obesity is a growing concern in obstetric practice and has been associated with an increased risk of adverse maternal and fetal outcomes [3]. The higher the BMI is, the higher the chance of undergoing LSCS [2]. It is worthwhile to know that BMI>50 kg/m^2^ comes under extreme obesity and poses a higher risk to both to the mother and babies [5]. Similarly, the BMI of our patient was above 50 kg/m^2^ and hence, there was a possibility of an adverse outcome if not delivered as soon as possible. A multidisciplinary team meeting involving anesthetic consultant, obstetrician consultant, trainees, patient, and the patient's mother was held swiftly within a short time and a decision to go ahead with LSCS was made. The Royal College of Obstetricians and Gynecologists (RCOG) also suggests a multidisciplinary approach involving a discussion between the consultant obstetrician, anesthetist, midwife, and the patient during the antenatal period regarding the mode of delivery [6].

Previous and recent studies found that in morbidly obese patients undergoing cesarean delivery, the incidence of intra-operative and post-operative complications including gestational diabetes, preeclampsia, hypotensive episodes, post-partum hemorrhage (PPH), preterm birth, stillbirth, neonatal seizures, and macrosomia were significantly higher than in non-obese patients [5-8]. Hence, morbidly obese patients require special consideration during labor, delivery, and post-partum care. Interestingly, one recent study reported that morbid obesity did not have any significant impact on PPH and it depended upon the mode of delivery (vaginal delivery increases the odds of having severe PPH by 19%) while LSCS decreased the odds by 14% of having severe PPH and thus in line with this study as this patient just had minor PPH [9]. Our patient did not develop any complications other than PPH.

The management of cesarean delivery in these patients presents several challenges, including technical difficulties, anesthesia issues, and post-operative complications. According to the American College of Obstetricians and Gynecologists (ACOG), cesarean delivery in obese women may require additional personnel and specialized equipment, including an operating room table with a higher weight capacity, longer instruments for abdominal wall retraction, and a larger surgical drape [10]. We also made the required arrangements which really helped us throughout the procedure.

Additionally, the guideline suggests that morbidly obese women should undergo pre-operative evaluation and optimization of comorbidities, including diabetes, hypertension, and obstructive sleep apnea [10]. In the present case, the patient was evaluated for all of the above and was found to be hypertensive which was known to us by her background for which her medications were optimized.

One of the significant concerns in morbidly obese patients is anesthesia management. Anesthesia in such patients can be challenging due to their altered anatomy, compromised respiratory function, and comorbidities. Various studies and guidelines have recommended regional anesthesia as a safe and effective option for morbidly obese patients undergoing LSCS. It is associated with lower risks of complications, such as pulmonary aspiration, mechanical ventilation, and cardiac arrest, compared to general anesthesia [6,10]. Similar to these guidelines, our anesthetic specialist decided to give a mixture of spinal and epidural anesthesia which was found to be beneficial for our patient.

With regards to incision, a very recent study conducted on patients who had recently given birth with an average BMI of 49 kg/m^2^ found that there was a tendency towards fewer wound complications when a high-transverse skin incision was used instead of a low-transverse incision (15.6% vs. 27.1%, with a p-value of 0.24). However, the group that underwent the high-transverse incision had lower 5-minute Apgar scores (8 vs. 9, with a p-value of 0.002) and a higher number of NICU admissions (28.1% vs. 5.2%, with a p-value of 0.001), but there was no significant difference in umbilical artery pH levels [11]. In order to avoid fetal complications, we went ahead with a lower transverse incision in our patient. The 5-minute Apgar score for our patient's child was 9 as compared to the average of 8 in the above study [11].

Post-operative management is another critical aspect of the care of morbidly obese patients undergoing cesarean delivery. These patients are at an increased risk of post-operative complications, such as wound infection, venous thromboembolism, and respiratory complications. ACOG and RCOG recommend that prophylactic anticoagulation should be considered in morbidly obese patients undergoing cesarean delivery, and early mobilization and deep breathing exercises should be encouraged [6,10]. The current patient was started on enoxaparin subcutaneously (S/C) for venous thromboembolism (VTE) prophylaxis.

Bariatric surgery is a possible treatment for severe obesity that does not respond to conventional medical methods. In this case, the woman was recommended to undergo bariatric surgery at the earliest feasible opportunity. It is recommended to wait for at least 12-18 months after undergoing bariatric surgery before attempting pregnancy to enable the stabilization of body weight and the detection and management of any potential nutritional deficiencies that may not be apparent during the initial months [6].

Recently, a study also reported a morbidly obese pregnant woman presenting with acute respiratory distress syndrome (ARDS) to the hospital suggesting ARDS as a complication as well. Unfortunately, the fetus also died in-utero. The patient was not aware of the pregnancy until a later stage of the second trimester and was not routinely followed up [12]. The pregnant woman in our care received consistent monitoring from her private obstetrician during her antenatal visits, which played a significant role in effectively managing her pregnancy and ensuring successful delivery, as any pre-existing medical conditions were controlled and no new complications arose. It is important to highlight the need for more frequent and specialized antenatal follow-ups for extremely obese patients due to their increased risk of complications.

To effectively manage cases of severely obese pregnant patients, it is essential to implement dedicated and regular antenatal monitoring, promptly address any co-existing medical conditions, adopt a multidisciplinary approach, ensure appropriate pre-operative preparation, use suitable anesthesia techniques tailored to the patient, and provide adequate post-operative care.

## Conclusions

In conclusion, managing LSCS in morbidly obese patients requires a multidisciplinary approach involving obstetricians, anesthetists, neonatologists, and nursing staff. The guidelines recommend pre-operative evaluation and optimization of comorbidities, specialized equipment, and regional anesthesia for cesarean delivery in obese women. Studies suggest that dedicated routine follow-ups, pre-conception counseling, optimizing glycemic control, and bariatric surgery may have long-term benefits in reducing the risk of adverse maternal and fetal outcomes in obese women. Further studies are needed to explore the optimal management strategies for cesarean delivery in obese parturients.
